# Health status of primary school educators in low socio-economic areas in South Africa

**DOI:** 10.1186/s12889-015-1531-x

**Published:** 2015-02-25

**Authors:** Marjanne Senekal, Zibuyile Seme, Anniza de Villiers, Nelia P Steyn

**Affiliations:** Division of Human Nutrition, Department of Human Biology, Faculty of Health Sciences, University of Cape Town, UCT Medical campus, Anzio Road, Anatomy Building, Floor 2, Room 2.04, Observatory 7925, Cape Town, South Africa; Chronic Diseases of Lifestyle Unit, Medical Research Council (MRC), Francie van Zyl Ave, Tygerberg, 7505 South Africa

**Keywords:** Educators, Teachers, Non-communicable diseases, Obesity, Hypertension, Diabetes

## Abstract

**Background:**

Non-communicable Diseases (NCDs) are major health concerns in South Africa. According to the life cycle approach NCD prevention strategies should target children. Educators are important external factors influencing behaviour of learners. The objective of this study was to assess the prevalence of selective NCD risk factors in educators of primary school learners.

**Methods:**

A cross-sectional design was used to assess the body mass index (BMI) and waist circumference (WC), blood glucose (BG), cholesterol (BC), blood pressure (BP), perceived health and weight, and parental NCD history of 517 educators in the Western Cape of South Africa.

**Results:**

The sample included 40% males and 60% females; 64% urban and 36% rural, 87% were mixed ancestry, 11% white and 2% black. Mean age for the total group was 52 ± 10.1 years, BMI 30 ± 1.2 kg/m^2^ (31% overweight, 47% obese), diastolic BP 84 ± 10.0 mmHg, systolic BP 134 ± 18.7 mmHg (46% high BP), BG 4.6 ± 2.3 mmol/L (2% high BG), BC 4.4 ± 0.9 (30.4% high BC) and WC 98 ± 14.1 cm for males (38% high WC) and 95 ± 15.3 for females (67% high WC). BMI was higher (p = 0.001) and systolic (p = 0.001) and diastolic (p = 0.005) BP lower in females. Rural educators were more obese (p = 0.001). BMI (p = 0.001) and systolic BP (p = 0.001) were lower in younger educators. Correct awareness of personal health was 65% for BP, 79.2% for BC and 53.3% for BG. Thirty-eight percent overweight/obese females and 33% males perceived their weight as normal.

**Conclusion:**

The findings of this study demonstrated a number of characteristics of educators in the two study areas that may influence their risk for developing NCDs and their potential as role models for learners. These included high levels of obesity, high blood pressure, high waist circumference, high cholesterol levels, and high levels of blood glucose. Furthermore, many educators had a wrong perception of their actual body size and a lack of awareness about personal health.

## Background

Globally non-communicable diseases (NCDs) are the major causes of mortality, with cardiovascular diseases, cancer, chronic lung diseases and diabetes being the leading ones [1]. More than 30 million people die annually from NCDs, with an estimated 80% of these occurring in low and middle income countries [2]. However, the majority of these diseases can be prevented through limiting exposure to the known shared risk factors such as unhealthy diets, physical inactivity, tobacco use and harmful use of alcohol [3,4].

The increasing prevalence of NCDs is thought to be largely attributable to insufficient development and implementation of strategies to reduce these diseases by national governments, civil society and international agencies [2,3]. Proven cost-effective strategies to prevent and control this growing burden are available, but high-level commitment and concrete actions are frequently missing at national level [1]. Limited resources in terms of funding for NCD prevention and control programmes is a further concern [5].

NCD prevention is not among the Millennium Development Goals despite the fact that it has a profound impact on the poorest people in low- and middle income countries and imposes a heavy burden on socio-economic development [4]. According to Mayosi et al. [5] prevention and treatment of NCDs have not been a priority in South Africa because of the high prevalence of communicable diseases such as HIV/AIDS and tuberculosis. There is however no doubt that NCD prevention should become a national health priority with interventions needing to focus on reducing the main modifiable risk factors for NCDs, namely tobacco use, unhealthy diets, physical inactivity and harmful use of alcohol [4]. As evidence points to the fact that modifiable risk factors emerge from childhood to adulthood [6], it is a matter of course that prevention strategies should target children at an early age.

The increasing prevalence of NCDs in South Africa and the need for early intervention motivated the Medical Research Council (MRC) to launch a primary school-based diabetes prevention longitudinal research study, HealthKick [7]. During the formative assessment phase of this study a situational analysis of a randomly selected sample of 100 primary schools (50 in the urban Metropole North education district and 50 in the rural Overberg/Breede River district) were conducted [7].

During the assessment phase of the study school principals identified lack of physical activity (33%) and NCDs (24%) as being main health priorities for educators [7]. The ecological approach followed during intervention planning, clearly identified educators as being a major potential environmental influence on the behaviours of learners, both at inter-personal level (as support to the learner and as possible role model in terms of health related behaviour) and at organizational level (as educator and potential programme implementer) [7]. This notion is supported by the work of Weinstein and Rosen [8] as well as the World Health Organization (WHO) [9], who reported that the educator is an important external factor influencing potential behaviour changes in learners. The importance of strengthening educators in this role has been demonstrated by Cargo et al.[10] who found that “educators would benefit from interventions that predispose, enable, and reinforce their capacity to adopt and apply health promotion roles”. With this in mind it was therefore decided to expand the formative assessment of the HealthKick study to include a health risk survey of educators involved with the target population, namely Grade four to six (10- to 12 year old) learners.

This paper reports on the prevalence of selective NCD risk factors (weight status, blood glucose, blood cholesterol, blood pressure), perceived NCD related health and weight status, NCD history in parents, as well as awareness of diabetes in this group of educators in an urban and a rural education district of the Western Cape Province. Perspectives on how this may influence the health related behaviour of learners are discussed.

## Methods

### Study design and sample

A cross-sectional, descriptive study design was used to assess the NCD risk indicators (weight status, blood glucose, blood cholesterol and blood pressure); perceived personal health and weight status, NCD history in parents of educators. Group comparisons were conducted to assess the association between key variables such as gender, age, weight status (BMI category) and origin (rural vs. urban).

The study population comprised of primary school educators from the Western Cape. The Western Cape Province Department of Basic Education (WCBE) has eight education districts (EDs), with four in rural areas and four are in urban areas. Two EDs, one in a rural setting (Overberg/Breede river) and one in an urban setting (Metropole North), were purposively selected for the situational analysis that included 100 schools, 50 in each area [7]. All male and female educators of grades 4 to 6 were invited to participate in the study. Educators from 83 of the original 100 schools included, participated in the study which took place in 2008, with the final sample including 329 educators from the rural and 188 educators from the urban settings, thus a total of 517 educators. Regarding the higher numbers in rural areas: The educators in the rural areas appeared to be keener to participate. This is probably due to the fact that there are far fewer opportunities and activities in the rural areas. They also appeared to be less busy and did not have to deal with issues like driving long distances to work and having problems such as gangs and violence to the same degree as those in urban areas.

### Anthropometric and clinical assessments

Anthropometric measurements included height and weight for the calculation of the body mass index (BMI), as well as waist circumference. Weight was measured on a digital CPW Plus 200 scale to the nearest 0.1 kg. Height was measured to the nearest centimetre with the educator standing on the base of a stadiometer facing outwards while the fieldworker lowered the sliding headboard until it was touching the cranium. Waist circumference was measured with an inflexible tape measure at the narrowest part of the abdomen. Two measurements were taken of each measure and the average was used.

BMI was calculated by dividing weight in kilograms by height in metres squared and categorised as healthy weight (18.5–24.9 Kg/m^2^), overweight (25.0–29.9 Kg/m^2^) or obese (BMI 30.0 Kg/m^2^ or more) [11]. Waist circumference was used to assess abdominal fat distribution and cardiovascular disease risk. This measure was interpreted using WHO cut-off points, with a waist circumference greater than 102 cm and 88 cm for men and women, respectively, reflecting increased risk [2].

An Omron 705CP automatic monitor was used to measure blood pressure. Three readings, two to three minutes apart, were taken and the first measurement was discarded. The second and a third measurements were averaged to provide the final value to be used. The blood pressure reading was interpreted using the cut-off values for South African adults, with a systolic measure greater or equal than 140 mmHg and or a diastolic pressure greater or equal than 90 mmHg considered to be high [12]. Individuals were not asked about anti-hypertensive medication, hence high BP measurements may have been underestimated.

Biochemical assessments included determination of blood glucose and total cholesterol levels using a random finger prick blood sample from each educator. The blood samples were analysed using ACCU-CHECK and ACCUTREND machines, respectively. A random blood glucose level of ≥11.1 mmol/l was considered to reflect abnormal blood glucose levels [13], while a blood cholesterol of more than 6.2 mmol/l indicated a high cholesterol level [13].

### Questionnaire

The questions for the structured self-administered questionnaire were developed using relevant literature, text books, existing questionnaires and input from expert panel members, three senior researchers with PhDs, two masters level and four honours level students). The questions included the assessment of socio-demographic variables (gender, age and ethnicity), personal health history [self-recalled prevalence of high blood pressure, heart attack, stroke, high cholesterol and diabetes (“told by a health professional or doctor”)], prevalence of these conditions in parents (as recalled by the educator); perception of current weight (normal weight, overweight or obese), as well as questions to assess awareness/perception of diabetes and the causes and symptoms of the disease.

### Data collection procedures

Schools were visited during three time slots depending on each school’s schedule, namely during first break, during second break or after school. Data collection stretched over a period of four months. While data collection took place on the school premises during and after school hours, investigators ensured that there was no interruption of normal academic activities in classes. After the purpose of the study and the methods to be used had been explained to the educators those who agreed to participate signed the consent form and were then assessed in a private school room. All the measurements and questionnaires were completed by each participant on the same day.

### Ethics

Ethical approval for the study was obtained from the University of Cape Town Faculty of Health Sciences Human Research Ethics Committee (REC REF: 486/2005). Permission to conduct the study in schools was obtained from the Western Cape Department of Basic Education. Principals of schools were subsequently approached with a letter that included information on the study, inviting them to participate. Informed written consent was obtained from educators before any tests were done.

### Data analysis

Descriptive statistics included frequencies for categorical variables and mean ± SD for normally distributed data and median (Interquartile Range) for non-normally distributed data, using the Shapiro-Wilk test to test for normality. Associations between BMI, gender, age, origin (rural or urban), waist circumference, biochemical and clinical indicators, personal and parental health history, perception of personal weight and awareness of diabetes were assessed using Pearson Chi square test for categorical comparisons and ANOVA (normally distributed data) or Kruskal-Wallis (non-normally distributed data) for continuous variables. Analyses were not adjusted for the cluster sampling frame.

## Results

### Socio-demographic profile of educators

The mean age for the total group was 52 years, with a significant difference between males and females (Table 1). When categorised according to age groups, it is evident that female educators were significantly more likely to be older than 50 years. Furthermore, the largest percentage of educators was of mixed ancestry (87%) and had urban origins (64%).

**Table 1:** **Socio-demographic profile of educators (n = 517)**

	**Total group (n = 517)**	**Male (n = 196)**	**Female (n = 321)**	**p-value**
**Age** (mean ± SD)	45 ± 7.9	44 ± 6.8	45 ± 8.6	*0.058* ^*1*^
**Age categories (%)**				
<30 years	2	1	2	*0.011* ^*2*^
30-49 years	72	80	68	
>50 years	26	19	30	
**Ethnicity (%)**				
African	2	1	3	*0.361* ^*2*^
White	11	10	11	
Mixed ancestry	87	89	86	
**Environment (%)**				
Urban	64	35	65	*0.067* ^*2*^
Rural	36	65	35	
**Total**	**100**	**38**	**62**	

^1^Pearson Chi-squared test.

^2^ANOVA.

### Anthropometric, biochemical and clinical profile

The mean BMI of the total group was in the obese range, with females and older educators having a significantly higher BMI than males and younger educators respectively (Table 2). Almost half (47%) of the educators were obese; 31% overweight and 21% normal weight. Female educators were significantly more likely to be obese and less likely to be a normal weight than male educators. Educators from urban areas were significantly more likely to be obese than those from rural areas. The likelihood of being obese also increased significantly with age, with 20% of those younger than 30 years being obese, compared to 58.5% of those older than 50 years who were obese.

**Table 2:** **Anthropometric measures and categorization according to cut-offs for the total group according to age, gender and origin groups (row %) (n = 517)**

		**Weight (kg)**	**Height (cm)**	**BMI (kg/m** ^**2)**^				**WC (cm)**		
**Category**	**n** ^**1**^	**Mean ± SD**	**Mean ± SD**	**Mean ± SD**	**% <25**	**% 25-29**	**%** ≥**30**	**Mean ± SD**	**% M** ≤ **102**	**M** > **102**
									**F** ≤ **88**	**F** > **88**
**Gender**										
Male	196	82 ± 2	170 ± 7	28 ± 5.6	28.0	37.0	35.0	98 ± 14.2	62.0	38.0
Female	321	80 ± 2	159 ± 2	31 ± 7.8	18.0	27.0	55.0	95 ± 15.3	33.0	67.0
*p-value*		*0.126* ^2^	*0.001* ^2^	*0.001* ^2^	*0.001* ^2^			*0.001* ^2^	*0.001* ^2^	
**Origin**										
Rural	329	80 ± 2	163 ± 2	29 ± 7.5	24.9	30.1	44.9	95 ± 14.8	46.2	53.8
Urban	188	82 ± 2	163 ± 8	31 ± 6.4	16.5	33.5	50.0	98 ± 15.0	40.9	59.0
*p-value*		*0.139* ^3^	*0.665* ^3^	*0.063* ^3^	*0.001* ^2^			*0.249* ^3^	*0.248* ^2^	
**Age categories**										
<30	10	69 ± 2	159 ± 6	27 ± 1.5	40.0	40.0	20.0	84 ± 18.0	70.0	30.0
30-49	366	81 ± 2	164 ± 2	30 ± 7.4	24.6	31.9	43.4	96 ± 15.0	48.9	51.1
>50	130	83 ± 2	162 ± 8	32 ± 6.3	13.9	27.7	58.5	99 ± 7.0	30.1	69.2
*p-value*		*0.053* ^3^	*0.372* ^3^	*0.001* ^3^	*0.001* ^2^			*0.001* ^3^	*0.001* ^2^	
**Total group**	517	81 ± 2	163 ± 2	30 ± 1.2	21.0	31.0	47.0	96 ± 15.0	44.0	56.0

BMI = Body Mass Index, WC = waist circumference, SD = standard deviation.

^1^n varies due to missing values.

^2^Pearson Chi-squared test.

^3^ANOVA; M = males; F = females.

When comparing actual weight classification according to BMI cut-offs, for which the overweight and obese categories were collapsed, with perceived weight (my weight is normal/I am overweight or obese) (results not shown in a table), it is evident that 96% (n = 55) of female educators in the normal weight category perceived their weight to be normal, while 4% (n = 3) thought that they were overweight/obese. Of the female educators in the overweight/obese category, 38.4% (n = 101) perceived their weight as normal, while 61.6% (n = 162) correctly thought that they were overweight/obese. The results for male educators shows that 85% (n = 47) of male educators in the normal weight category perceived their weight to be normal, while 15% (n = 8) thought that they were overweight/ obese. Thirty two point six percent of male educators in the overweight/obese category perceived their weight as normal, while 67.4% (n = 96) correctly thought that they were overweight/ obese.

The mean waist circumference in males was significantly higher than that in females. The mean value for females was above the cut-off that indicates increased health risk, while this was not the case for males (Table 2). This is also reflected by the fact that almost two thirds of the females had a waist circumference above the cut-off, compared to just above a third for males. The mean waist circumference increased significantly with age, as did the likelihood to have a waist circumference above the cut-off.

The mean diastolic and systolic blood pressure of the total group was below the respective cut-offs for hypertension [12] (Table 3). When classified according to blood pressure categories, almost half of the educators fell in the hypertension category, with female educators, those from rural origins and those in the 30–49 year age group being significantly more likely to have hypertension. The >50 years group were the least likely to have hypertension.

**Table 3:** **Biochemical and clinical measures and categorisation according to cut-offs for the total group and for age, gender and origin and groups (n = 517)**

		**BP (mmHg)**				**Glucose (mmol/l)**				**Cholesterol (mmol/l)**		
		**Mean ± SD**		**%** **<** ^**140**^ **/**_**90**_	**%** ≥^**140**^ **/**_**90**_	**Mean ± SD**	**%** **<5.6**	**%** ≥**5.6-11.1**	**%** ≥**11.0**	**Mean ± SD**	**%** **<5.2**	**%** **>6.2**
**Category**	**N** ^**1**^	**Diastolic**	**Systolic**									
**Gender**												
Male	196	86 ± 10.9	137 ± 18.4	63.0	37.0	4.99 ± 2.4	65.8	31.6	2.6	4.5 ± 0.8	64.8	35.2
Female	321	83 ± 10.0	131 ± 18.6	50.0	50.0	4.44 ± 2.4	74.0	24.0	1.6	4.5 ± 0.9	72.6	27.4
*p-value*		*0.005* ^3^	*0.001* ^3^	*0.003* ^2^		*0.074* ^3^	*0.121* ^2^			*0.062* ^3^	*0.062* ^2^	
**Origin**												
Rural	329	85 ± 10.3	132 ± 18.5	50.2	49.8	4.7 ± 2.2	72.9	25.5	1.5	4.6 ± 0.9	67.8	32.2
Urban	188	84 ± 10.6	135 ± 18.9	62.2	37.7	4.6 ± 0.9	67.5	29.8	2.7	4.3 ± 0.8	72.7	27.3
*p-value*		*0.410* ^3^	*0.237* ^3^	*0.008* ^2^		*0.805* ^3^	*0.351* ^2^			*0.227* ^3^	*0.240* ^2^	
**Age categories**												
<30	10	80 ± 7.9	122 ± 13.6	60.0	40.0	4.3 ± 1.7	70.0	30.0	0.0	3.9 ± 0.5	80.0	20.0
30-49	366	84 ± 10.7	132 ± 18.0	49.7	50.3	4.5 ± 2.1	74.0	24.3	1.6	4.4 ± 0.8	70.5	29.5
>50	130	84 ± 9.4	139 ± 20.0	68.5	31.5	5.2 ± 2.5	63.1	34.6	2.3	4.6 ± 0.9	66.2	33.9
*p-value*		*0.365* ^3^	*0.001* ^3^	*0.001* ^2^		*0.517* ^3^	*0.212* ^2^			*0.504* ^3^	*0.502* ^2^	
**Total group**	**517**	**84 ± 10.0**	**134 ± 18.7**	**55.0**	**46.0**	**4.65 ± 2.3**	**71.0**	**27.0**	**2.0**	**4.47 ± 0.85**	**69.6**	**30.4**

BP = blood pressure, SD = standard deviation.

^1^n varies due to missing values.

^2^Pearson Chi-squared test.

^3^ANOVA.

From Table 3 it is evident that the mean random blood glucose level for the total group was within the normal range, with no significant differences between any of the groupings (gender, origin, age). About a third (27%) of the educators had a random blood glucose level in the high normal range and only 2% were in the diabetes risk range. The mean random cholesterol level for the total group was also within the normal range, with no significant differences between any of the groupings (gender, origin, age). Almost a third (30.4%) of the total group had a random cholesterol level in the high range.

### Actual and recalled personal health status and perceived parental health status

Results on the self-recalled prevalence of particular conditions (“told by a health professional or doctor”) for educators as well as for their parents (as recalled by the educators), are presented in Table 4. The most common condition that educators reportedly had was high blood pressure (33.3%), followed by high cholesterol (13%) and diabetes (12%). The most common condition reported for mothers and fathers was also high blood pressure (51% and 26% respectively). Diabetes was the second most common condition reported for mothers, while a heart attack was second most common condition reported for fathers.

**Table 4:** **Personal** ^**1**^ **and parental disease** ^**2**^ **history for the total group (column %) (n = 512** ^**3**^ **)**

**Disease/condition**	**Personal %**	**Mother %**	**Father %**
**High blood pressure**			
Yes	33.3	51.3	25.9
No	61.7	38.1	57.2
Unsure	5.0	10.7	16.9
**Heart attack**			
Yes	1.2	10.1	19.6
No	96.9	83.2	68.6
Unsure	2.0	6.7	11.9
**Stroke**			
Yes	2.2	13.3	11.9
No	96.5	81.7	76.5
Unsure	1.4	5.0	11.5
**High cholesterol**			
Yes	13.0	13.8	9.6
No	82.5	67.1	69.1
Unsure	4.5	13.0	21.3
**Diabetes/sugar**			
Yes	11.6	26.2	16.1
No	85.7	66.3	68.8
Unsure	2.6	7.5	15.1

^1^“told by a health professional or doctor”.

^2^As recalled by the educators.

^3^n varies due to missing values.

Table 5 presents a comparison between educators’ actual health status based on non-fasting blood glucose, cholesterol levels, and blood pressure, compared with self-recalled conditions. Almost a third (28.8%) of those with high blood pressure and half of those with high glucose levels did not recall having been told by a health professional that they had these conditions. The majority (83.9%) of those with normal cholesterol values recalled having been told by a health professional that they had this condition.

**Table 5:** **Educators’ actual health status compared to self-reported diseases** ^**1**^ **for the total group (column %) (n = 512** ^**2**^ **)**

	**Actual health status**					
	**Blood pressure**		**Cholesterol** ^**3**^		**Glucose** ^**3**^	
**Recall having been told by a health professional or doctor**	**Normal**	**High**	**Normal**	**High**	**Normal**	**High**
	**n = 279**	**n = 233**	**n = 354**	**n = 154**	**n = 493**	**n = 15**
Normal	37.0	28.8	11.9	15.6	10.6	46.7
High	58.7	65.7	83.9	79.2	86.6	53.3
Unsure	4.7	5.6	4.2	5.2	2.8	0.0

^1^Agreement not compared statistically as actual health status does not include an “unsure” category.

^2^n varies due to missing values;^3^Random blood sample.

### Awareness/understanding of diabetes

Table 6 presents data on awareness of factors contributing to diabetes as well as the causes and symptoms of diabetes. Almost all educators mentioned fatty foods as a cause, three quarters mentioned obesity and 57.7% mentioned sugar/sweets. Males were significantly more likely than females to think that sugar and sweets caused diabetes, with those from urban origins also being significantly more likely than those from rural origins to think that sugar and sweets caused diabetes. All the educators younger than 30 thought that sugar and sweets caused diabetes, while only half of those older than 30 thought this. The most commonly known sign of diabetes in the total group was slow healing of wounds (80.6%), followed by being thirsty (81.6%), weight loss (73.1%), blurred vision (72.7%), peeing all the time (60.5%), constant hunger (52.5%) and repeated infections (42.5%). Vomiting was not a commonly known sign of diabetes. There were no significant differences between gender, BMI, origin and age groups for these variables. Although not significant, teachers younger than 30 tended to be less aware of all eight signs of diabetes included in the questionnaire.

**Table 6:** **Awareness/understanding of causes and symptoms of diabetes for the total group and by gender, origin, age and BMI groups**

		**Contributing to diabetes** ^**2**^				**Symptoms of diabetes** ^**2**^							
**Category**	**N** ^**1**^	**Sugar & sweets**	**Super natural**	**Obesity**	**Fatty foods**	**Being very thirsty**	**Peeing all the time**	**Vomiting**	**Weight loss**	**Constant hunger**	**Slow healing if wounds**	**Blurred vision**	**Repeated infections**
**Gender**													
Male	196	65.6	10.0	79.7	90.7	76.7	61.7	11.3	71.6	46.9	77.3	71.7	42.1
Female	321	57.8	12.5	73.3	90.3	84.5	59.8	10.4	74.0	55.9	82.6	73.3	42.8
*p-value* ^3^		*0.005*	*0.393*	*0.105*	*0.878*	*0.026*	*0.679*	*0.751*	*0.567*	*0.049*	*0.144*	*0.679*	*0.877*
**Origin**													
Rural	329	55.4	13.4	75.9	90.5	80.5	60.9	11.2	68.9	52.5	80.8	74.6	41.1
Urban	188	61.6	8.2	75.5	90.2	83.4	59.9	10.2	80.2	52.4	80.2	69.4	45.1
*p-value* ^3^		*0.007*	*0.111*	*0.408*	*0.237*	*0.411*	*0.828*	*0.745*	*0.006*	*0.986*	*0.871*	*0.20*	*0.377*
**Age categories**													
<30	10	100.0	20.0	80.0	80.0	66.7	30.0	0.0	50.0	40.0	70.0	40.0	40.0
30-49	366	58.8	9.5	76.6	91.5	79.8	60.8	11.6	72.3	53.6	79.8	73.6	43.1
>50	130	50.8	15.8	70.9	87.5	86.7	59.4	9.4	74.8	50.0	83.4	72.7	38.6
p-value^3^		0.007	0.111	0.408	0.237	0.145	0.419	0.419	0.236	0.570	0.448	0.063	0.671
**BMI**													
Normal	113	55.1	7.3	72.5	87.2	72.1	51.5	9.0	68.5	45.9	74.8	63.1	41.4
Overweight	162	62.4	11.7	77.6	89.2	83.1	64.6	10.7	72.2	56.1	76.1	73.4	40.8
Obese	242	55.7	13.3	76.1	92.7	84.9	62.1	11.7	77.1	56.1	86.3	76.7	44.3
*p-value* ^*3*^		*0.347*	*0.272*	*0.629*	*0.219*	*0.013*	*0.073*	*0.756*	*0.080*	*0.203*	*0.009*	*0.028*	*0.763*
**Total**	517	57.7	11.5	75.8	90.4	81.6	60.5	10.8	73.1	52.5	80.6	72.7	42.5

^1^n varies due to missing values.

^2^% YES depicted, the balance responded NO, row %.

^3^Pearson Chi-squared test.

## Discussion

The socio-demographic profile of educators who participated in our research shows that they were mostly from mixed ancestry and between 30 and 49 years of age, with a third being 50 years or older. The profile indicates that the educators in the present study may have been on average older than the national profile i.e. 21% of South African educators being under the age of 40 years, 36% being between 40 and 50 years and only 12% being between 50 and 60 years [14]. Approximately a third of our sample were male and two thirds female, which is in line with the gender profile indicated in the South African Country Report [15].

The prevalence of overweight and obesity in the educators in the present study is far above the prevalence reported in adults in the 2012 national South African Nutrition and Health Survey (SANHANES), for males, being19.6% and 11.6% respectively; and females 25% and 40.1% respectively, for all race groups [16]. As is also evident from national data [16], the prevalence of obesity was significantly higher in female educators, increased with age, and was also higher in those living in urban areas. Waist circumference measures, indicative of central adiposity, show that two thirds of female educators compared with 50% in SANHANES and just more than a third of male educators compared with 10% in SANHANES had a waist circumference in the risk range. The increase in waist circumference with age found in our study is also in line with national data [16]. With the high prevalence of obesity and the added risk of central adiposity, it could be argued that both male and female educators in the study have a higher risk of developing NCDs than the general population.

According to the WHO [17] obesity is one of the four key metabolic/psychological changes that occur in response to an unhealthy lifestyle. The other three changes are raised blood pressure, hyperglycaemia and hyperlipidaemia, with overweight and obesity being possible risks for these three metabolic/physiological changes [17]. It is thus not surprising that hypertension, high blood cholesterol and high glucose levels were found in our sample of educators.

Nearly half (46%) of educators in the present study had hypertension at the time of study. This prevalence was far above that found in the SANHANES 2012, namely 10.2% of males and females and 11.8% of those of mixed ancestry having hypertension. Educators in our study who were younger than 30 as well as those older than 50, were significantly less likely to have high blood pressure. The latter may reflect compliance with medication in the older group (not assessed in this research), while the higher blood pressure in the 30–50 year olds may reflect lack of awareness and thus treatment of high blood pressure (see discussion on health awareness below). Educators from the rural areas were more prone to have a high blood pressure, which was not expected, as they were significantly less likely to be overweight/obese.

About a third (30.4%) of the sample had high blood cholesterol (random blood sample), and 29% had high glucose levels. The prevalence of high cholesterol in this study far exceeds the 18.9% found in SANHANES [16]. Of note is the fact that hypertension, diabetes and heart disease (heart attack) seemed to be very prevalent in the mothers and/or fathers of the educators, reflecting a possible genetic susceptibility to these NCDs.

When considering educators’ perceptions of their health status, it is clear that they are not necessarily aware of having specific health problems. A third of those who had hypertension at the time of the study, reported never having been told so, which is in line with the findings by Thankappan et al.[18] in a community-based study in India (36.9% of people with hypertension were aware of their condition) but much lower than the 74% reported awareness of the participants in the NHANES (1999–2010) [19]. Women, in the study by Thankappan et al. [18] were more likely to be aware of having hypertension. The fact that 58.7% of educators with normal blood pressure levels at the time of our study reported that they had been told by a health professional that they had high blood pressure, may be explained by either poor awareness/understanding of hypertension as such, or possible compliance with medical management of the condition by these individuals subsequent to diagnosis by a health professional. Awareness of high glucose levels was better than awareness of high blood pressure, in both educators who participated in our study (53.3%) and adults in Kerala State, India (72%) [18] and comparable to that in the United States [20]. This may be due to a perception that high blood pressure is a less serious health problem than high blood glucose (diabetes). Educators’ perceptions regarding having high cholesterol levels and having been told so by a health professional were most accurate. These observations emphasize the importance of public health education to increase awareness of and combat the increasing burden of NCD risk factors.

In line with the seemingly poor awareness of personal health problems, is the finding that 38.4% of the female educators and 32% male educators who were overweight/obese, thought that their weight was normal. Similar findings were reported by the Demographic and Health Survey in 2002 [21], namely that more than half of African women were overweight or obese, however, only 27% perceived themselves as being overweight. This may reflect poor general awareness of overweight/obesity, or poor diagnosis thereof at health care facilities or cultural/social acceptance of a larger body size as the norm, or a combination of these possibilities.

The literature shows that cultural influences are strongly linked to the acceptance of a larger body size in black women, as it is equated with being well cared for by the husband and reflects affluence and happiness [22-24]. However, it is not known to what extent cultural norms of a larger body size determine body size ideals in those of mixed ancestry. It could be argued that with such a high prevalence of overweight/obesity as was found in this research, social acceptance in the workplace as well as in the family/community of overweight/obesity as a norm may be a reality, exposing learners to overweight/obesity as a norm.

The apparent lack of awareness of health status in educators is a concern for various reasons, including the possibilities that it may reflect lack of interest in health as such, and thus a possible lack of interest in the need to change their lifestyle, as well as not seeking treatment for obesity, hypertension and metabolic indicators of NCDs. Findings by Misra and Khurana [25] in their study done in South Asians and Caucasians, namely that there is an association between lack of health awareness, negligence, inappropriate health education and poor adherence to treatment, also support the need to be concerned about the possible negative effects of lack of health awareness on health outcomes.

International research indicates that lack of knowledge/awareness of health status can be addressed by appropriate interventions. Fezeu et al.[26] showed in Cameroon that 80% of 1000 participants achieved a Global Diabetes awareness score that reflected good awareness/understanding of the risk factors, symptoms, treatment and complications of diabetes. The authors indicate that this outcome may have been the result of a health promotion and education on diabetes programme implemented in the area over a period of four years. Zhang et al. [27] found that only 43% of an urban sample of Chinese knew that obesity and 25% that inactivity were risks for diabetes. These figures improved to 85% and 76% respectively following the implementation of a multimedia health promotion programme. However, Guo et al.[19] reported poor improvement in awareness of hypertension in adults in the United States, despite a heavy campaign of programmes, guidelines, and policies to facilitate improved prevention, awareness, diagnosis, treatment and control of hypertension.

Despite the lack of awareness of their own health problems, it needs to be noted that almost all educators had heard of diabetes at the time of the study. Educators also seemed to be aware of important causes and symptoms of diabetes. The majority knew that obesity could cause diabetes. The fact that more than 90% identified fatty foods as being a cause of diabetes may reflect the possibility that the South African Food Based Dietary Guideline “eat fat sparingly” is “heard” by the population. There is on-going debate as to the role of sugar in the development of diabetes [28], but our results show that there is a strong perception amongst educators that sugar and sweets are a cause of diabetes. Male educators, participants from the urban area and those younger than 30 were significantly more likely to hold this opinion.

There is no doubt that educators play an important role in health promotion efforts aimed at children in the school setting, as they teach the official curriculum and in the process have the potential to create a supportive environment [29]. Educators are furthermore often expected to be intervention programme implementers and may also serve as role models to learners for healthy behaviours [9,30,31]. The WHO School Policy Framework [9], thus highlights the importance of the need for educators to be aware of and be responsible for the messages they give to learners as educators, potential programme implementers and as role models.

The view of educators as role models has been seriously touted since the eighties when it was proposed that health messages increase in effectiveness when they are promoted by teachers practicing healthy behaviours [32]. A number of characteristics of teachers in the two study areas that may influence their role as educators, potential intervention implementers and role models are evident. The high ratio of female teachers may reflect the presence of a supportive environment in many of the schools, as female educators have been reported to be more supportive than male educators [33]. However, the fact that the educators were older than the national profile, may reflect less enthusiastic engagement with education [34] . As per the suggestion by Vander Schee and Gard [32], namely that unhealthy teachers are less effective in the classroom, the high obesity and hypertension prevalence in the study population may affect quality of education and potential intervention implementation. The lack of awareness of being overweight/obese and having hypertension, high blood glucose and cholesterol levels may preclude teachers from understanding the need to address modifiable risks for these problems and/or receiving treatment. The very high prevalence of overweight/obesity in the educators and their apparent lack of awareness thereof is a specific concern. As mentioned, this may reflect social acceptance of overweight/obesity as a norm, which could be accepted by learners as the norm, possibly promoting early establishment of overweight/obesity.

It is believed that improving the health of educators also improves their effectiveness as role models for their students. Students are more likely to eat healthier foods if their educators are of normal weight and are regularly seen to eat healthy foods compared with obese educators who snack on unhealthy high energy foods [33]. Despite the limitations of the study (voluntary sample; no adjustment for the cluster sampling frame in analyses, self-reported information on health status of self and parents; and limitations of the finger prick method of analysing blood levels of glucose and cholesterol) it appears that lifestyle intervention for educators may be necessary to ensure that educators serve as a positive environmental influence for the entrenchment of healthy lifestyle behaviours in learners.

Recommendations for improving the health of educators should include the following:

At school, local and national level relevant policies need to be developed and effectively implemented to guide and facilitate interventions aimed at improving the health of educators. Such interventions should target dietary intake, physical activity and smoking behaviours of, as well as lack of health awareness, perception of weight status and relevant knowledge among educators, to be developed and implemented to improve their health and well-being and ensure that they serve as a positive environmental influence for the entrenchment of healthy lifestyle behaviours in learners. For educators to be and remain effective practitioners they should be enabled to “adopt enabling wellbeing strategies” from the outset, thus at the point of first appointment as educator. Furthermore, emphasis should be placed on the creation of a supporting food environment, such as types of foods sold at schools, as educators spend a large part of their day in this environment.

## Conclusions

The findings of this study demonstrated a number of characteristics of educators in the two study areas that may influence their role as educators and potential implementers of interventions. These included high levels of obesity, high blood pressure, high waist circumference, high cholesterol levels, and high levels of blood glucose. Furthermore, many educators had a wrong perception of their actual body size and a lack of awareness about personal health. The NCD risks factors found to be common among primary school educators calls for an urgent need for policy makers to implement health policy and interventions aimed at alleviating such risks in order to reduce the development of NCDs in this high risk group and ensure quality education of learners, intervention implementation and appropriate role modelling.
